# Causes of death in people living with HIV from a North London cohort between 2006 and 2023: A descriptive analysis

**DOI:** 10.1111/hiv.70054

**Published:** 2025-06-04

**Authors:** Linda Cheyenne Vaccari, Damien K. Ming, Jane Hazell, Alan Hunter, Fiona M. Burns, Robert F. Miller

**Affiliations:** ^1^ HIV Services Royal Free Hospital, Royal Free London NHS Foundation Trust London UK; ^2^ Centre for Antimicrobial Optimisation Imperial College London London UK; ^3^ Institute for Global Health, University College London London UK

**Keywords:** AIDS, cause of death, CoDE, HIV, infection, malignancy

## Abstract

**Background:**

The provision of highly active anti‐retroviral therapy has improved outcomes for people with HIV, worldwide. There are few data on trends and changes in the cause of death among people with HIV in the United Kingdom since its advent.

**Methods:**

We retrospectively reviewed deaths in people attending HIV services at Royal Free Hospital London between 2006 and 2023. Cause of death was categorized using the CoDe protocol. Analysis included description of demographics over time, HIV‐specific metrics (late diagnoses, AIDS‐defining illnesses) and aspects related to HIV treatment and trends in non‐AIDS‐related causes of death.

**Results:**

Of 529 deaths, 79.8% were male. Cause of death was non‐AIDS‐defining malignancy 21.4%, non‐AIDS‐defining infection 12.1%, AIDS‐defining infection 11.2%, AIDS‐defining malignancy 7.8%, self‐harm 9.3%, cardiovascular 8.3%, liver 2.8%, respiratory 2.6%, other 7.2% and unknown 17.4%. Comparing 2006–2011 and 2018–2023, the proportion of those dying from AIDS‐defining infection and malignancy fell from 13.8% to 7.1%, and from 13.8% to 3.1%, respectively; median age at death increased from 44.9 years (interquartile range [IQR] 39.7–52.4) to 58.0 (IQR 52.0–67.7): *p* < 0.001 and median interval between HIV diagnosis and death increased from 8.5 years (IQR 2.9–14.0) to 19.1 (IQR 11.8–26.1): *p* < 0.001.

**Conclusions:**

Between 2006 and 2023, there was a significant increase in median age at death and in the interval between HIV diagnosis and death. The proportion of deaths associated with AIDS‐defining infection and malignancy fell, while non‐AIDS‐defining infection, malignancy and deaths from self‐harm increased. These data suggest that focusing on earlier diagnosis, holistic clinical management and support for mitigating modifiable lifestyle risk factors including cancer screening and mental health services could result in improved outcomes and reduce preventable deaths.

## INTRODUCTION

The availability of effective anti‐retroviral therapy has improved outcomes for people living with HIV worldwide. In the United Kingdom, an estimated 107 000 people were living with HIV in 2023, and the number of deaths in this population has decreased from >1500 to 300 per annum between 1995 and 2023 [1].

The Ian Charleson Day Centre (ICDC) delivers care to over 3000 adults living with HIV and is based at the Royal Free Hospital (RFH), a university‐associated inner London NHS care provider. In 2006, a site‐specific database was established to record details of deaths among people receiving clinical care at RFH. From 2012, deaths in people with HIV in London (including RFH) were reported to the NHSE/PHE London HIV Mortality Review Group [2]: since 2019, deaths reported from RFH have been included in the UKHSA/BHIVA National HIV Mortality Review [3].

Surveillance systems, including the HIV and AIDS Reporting System (HARS), have been essential in understanding the changing epidemiology of HIV in the United Kingdom [4]. Accurate characterization of mortality data, including the contribution of non‐communicable diseases and co‐infections, is crucial for understanding and addressing the drivers of premature mortality. However, in the United Kingdom, there is poor understanding of temporal trends in the cause of death or change in patient demographics among people with HIV regarding their causes of death [5].

We retrospectively reviewed deaths in people attending HIV services at RFH over an 18‐year period. The aim of analysing this longitudinal cohort was to describe temporal trends in cause of death and to identify aspects of clinical care provision which could be targeted to improve outcomes.

## METHODS

This retrospective review was done at a single HIV treatment centre in North Central London, UK. People who were accessing HIV care at the RFH and who died between 11 February 2006 and 4 December 2023, in RFH or at another hospital, at home, in a hospice and those whose place of death was unknown (including deaths abroad) were included. Details were extracted from a local registry and the death certificate, with additional socio‐demographic and clinical information obtained from medical record linkage. Modifiable comorbidity data that could be easily extracted from laboratory results, including hepatitis B/C co‐infection and diabetes, were included.

For each person, a cause of death was independently attributed using the Coding Causes of Death in HIV (CoDe) protocol [6] by two infectious diseases specialists (LCV and DKM) to obtain a consensus. Disagreements were resolved through discussion with a third clinician (RFM), including individuals where the cause of death was incompletely described or unavailable.

Death was coded as AIDS‐related if the person had an AIDS‐defining condition, as per CDC definitions.

The analysis describes the demographics of the cohort over time as well as HIV‐specific metrics (proportion of late diagnoses and AIDS‐defining illnesses), aspects related to HIV treatment and trends in non‐AIDS‐related causes of death.

All data were anonymized. Descriptive data analyses were performed in Python 3.7 (Python.org) using Mann–Whitney tests for non‐parametric continuous variables and Chi‐squared tests for categorical variables; *p* < 0.05 was regarded as significant. A rolling window analysis was used to compare variables across three 6‐year groups.

Late, and very late, diagnoses were defined as CD4 count ≤350 and ≤200 cells/μL, respectively, at diagnosis. HIV viral suppression was defined as ≤200 copies/mL in blood.

This work was registered as a Clinical Audit with the Royal Free London NHS Foundation Trust (RFH site).

## RESULTS

**FIGURE 1:**
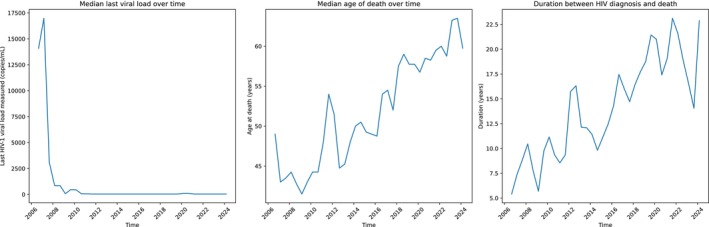
Rolling window analysis for number of deaths, age at death and time between diagnosis and death, 2006 to 2023.

Of all 529 deaths, 422 (79.8%) were in males and 197 (37.2%) were in people of White British ethnicity (Table 1). The number of recorded deaths peaked around 2007 and again in 2022 (Figure 1). The median age at diagnosis increased from 30 years for those diagnosed in the 1980s to 35, 42, and 48 years for those diagnosed in the 1990s, 2000s or after 2010, respectively. The median age at death across the cohort was 52.0 years (interquartile range [IQR] 43.8–61.2), increasing significantly between 2006–2011 and 2018–2023; *p* < 0.001 (Table 1).

**TABLE 1:** Demographics, factors related to clinical care, causes of death and trends over the course of the 18‐year cohort.

Category	All patients	Period		*p* Value
		1/1/2006–31/12/2011	1/1/2018–31/12/2023	
*N*	529	188	196	
Median age at death (years)	52 (43.8–61.2)	44.9 (39.7–52.4)	58.0 (52.0–67.7)	<0.001
Male sex	80.1% (399/498)	76.8% (139/181)	83.8% (145/173)	1.000
Ethnicity				
White British	39.6% (197/498)	34.3 (62/181)	42.8% (74/173)	0.380
Black or Black British	19.7% (98/498)	23.2 (42/181)	14.5% (25/173)	0.019
Any other White background	12.9% (64/498)	9.9% (18/181)	15.6% (27/173)	0.260
Any other Black background	5.0% (25/498)	5.5% (10/181)	5.2% (9/173)	0.900
Other	22.9% (114/498)	27.1% (132/181)	22.0% (135/173)	0.460
Median CD4 at diagnosis (cells/uL)	225 (81–447)	205 (58–403)	264 (94–502)	0.070
Late HIV diagnosis	60.6% (321/529)	64.9% (122/188)	54.0% (106/196)	0.040
Median duration between diagnosis and death (years)	13.2 (6.6–29.3)	8.5 (2.9–14)	19.1 (11.8–26.1)	<0.001
Median HIV‐1 viral load nearest to death (copies/mL)	40 (40–317)	54 (40–22 214)	40 (40–40)	<0.001
Place of death (*n*)				
Hospital	44.2% (234/529)	46.8% (88/188)	43.9% (86/196)	1.000
Intensive care	10.8% (57/529)	12.2% (23/188)	9.7% (19/196)	0.500
Home	22.1% (117/529)	11.7% (22/188)	24.5% (48/196)	0.002
Hospice	8.1% (43/529)	6.9% (13/188)	9.2% (18/196)	0.500
Unknown	25.1% (133/529)	34.6% (65/188)	22.4% (44/196)	0.010
Causes of death				
Non‐AIDS malignancy	21.4% (113/529)	19.7% (37/188)	26.5% (52/196)	0.150
Non‐AIDS infection^a^	12.1% (64/529)	8.0% (15/188)	18.4% (36/196)	0.005
AIDS infection	11.2% (59/529)	13.8% (26/188)	7.1% (14/196)	0.050
AIDS malignancy	7.8% (41/529)	13.8% (26/188)	3.1% (6/196)	<0.001
Self‐harm^b^	9.3% (49/529)	5.3% (10/188)	11.2% (22/196)	0.060
Cardiovascular^c^	8.3% (44/529)	3.2% (6/188)	10.7% (21/196)	0.008
Liver (excl. chronic alcohol excess)	2.8% (15/529)	4.8% (9/188)	1.6% (3/188)	0.120
Respiratory	2.6% (14/529)	2.7% (5/188)	2.0% (4/196)	0.940
Other	7.2% (38/529)	5.3% (10/188)	7.1% (14/196)	0.627
Unknown	17.4% (92/529)	22.9% (43/188)	12.2% (24/196)	0.009

Abbreviations: CVA, cerebro‐vascualar accident; IQR, interquartile range.

^a^ COVID‐19 was the cause of death for 10/36 of non‐AIDS infection deaths between 2018 and 2023.

^b^ Self‐harm causes of death include suicide, acute intoxication and chronic alcohol excess.

^c^ Cardiovascular causes of death include CVA, myocardial infarction and other ischaemic heart disease including heart failure.

The proportion of people with a late HIV diagnosis based on CD4 criteria decreased from 64.9% (122/188) in 2006–2011 to 54% (106/196) in 2018–2023; *p* = 0.04 (Table 1).

The median duration between HIV diagnosis and death over the 18 years increased from 8.5 years (IQR 2.9–14.0) in 2006–2011, to 19.1 years (IQR 11.8–26.1) in 2018–2023; *p* < 0.001 (Table 1).

Data from those dying after 2017 showed that of 221 people, at the time of death, 27% (106/221) had diabetes mellitus, and of 187 where information was available, 6.4% (12/187) had hepatitis B and 19.3% (36/187) had hepatitis C.

### Factors related to clinical care

Of those who died, 82.7% (438/529) people attended ICDC within the year before death. The median CD4 count at diagnosis for all people who died was 225 cells/μL (IQR 81–447).

Among all people, last viral load was measured a median of 70 days prior to death (IQR 26–165). When last measured, 27% (133/497) had a viral load >200 copies/mL. The proportion of people who were virologically suppressed (VL < 200 copies/mL) remained similar over time; 71.3% (134/188) in 2006–2011 and 68.4% (134/196) in 2018–2023.

A date of diagnosis was available for 500 of 529 people. Overall, 10.6% (53/500) died within 12 months of HIV diagnosis. These people tended to be younger (44 vs. 53 years; *p* < 0.001) and had a lower initial CD4 count (49 vs. 269 cells/μL, *p* < 0.001) compared with those who died later. Of those dying within 12 months of diagnosis, 61% (31/51) had a viral load >200 copies/mL prior to death. Of all deaths within 1 year, 60% (32/53) occurred between 2006 and 2011, 30% (16) between 2012 and 2017 and only 9% (5) between 2018 and 2023.

Reviewing antiretoviral (ARV) prescription data, the last script date was available for 433 people; 348 (80%) had received a script for ARVs within 6 months before death; however, 52 people (12%) had not had a script for ARVs in the year prior to death.

Hospital was the place of death for 44.2% (*n* = 234), with almost a quarter (24.4%) following an admission to intensive care. The proportion of people who died at home increased from 11.7% (22/188) to 24.4% (48/196) between 2006–2011 and 2018–2023, respectively; *p* = 0.002 (Table 1).

### Causes of death and trends

We were able to categorize 83% (437/529) of the deaths using the CoDe methodology. Information was insufficient to code the cause of death for 17% (92/529); people in this group were more likely to be younger and male. Notably, 35% (32/92) had a detectable VL (>200 copies/mL) when last recorded, 35% (32/92) had not had an ARV script within 6 months before death and 11% (10/92) died within 12 months of diagnosis. At least 8 of 92 unclassified deaths occurred abroad.

Causes of death were: non‐AIDS‐defining malignancy (21.4%), non‐AIDS‐defining infections (12.1%), AIDS‐defining infection (11.2%), self‐harm including intoxication, suicide, substance abuse (9.3%) and AIDS‐defining malignancy (7.8%): 5.7% of deaths were related to cardiovascular disease, 3.8% to chronic liver disease (including chronic alcohol misuse, HBV or HCV), 2.6% to respiratory disease and 2.5% to stroke (Table 1).

There were 100 deaths due to AIDS‐defining conditions: these were more likely to occur in people who were younger (70% <52 years old), male (62%) and of black ethnicity (41%). Almost a third (32%) of the AIDS‐defining conditions causing death occurred within 12 months of diagnosis. Of those whose death was AIDS‐defining, 52% had a detectable viral load (>200 copies/mL) when last checked, although 83% had received an ARV script within 6 months before death. Half (52%) of AIDS‐defining deaths occurred between 2006 and 2011, 28% between 2012 and 2017 and 20% between 2018 and 2023. The 59 deaths due to AIDS‐defining infections were most commonly caused by mycobacterial infection, including TB (12), AIDS itself (11) and PML (7), with five cases each of HIV encephalitis, cryptococcal meningitis, PCP and invasive fungal infections.

AIDS‐defining malignancy was the cause of death in 41 people (non‐Hodgkin lymphoma [*n* = 37, 90.2%], Kaposi sarcoma [*n* = 4, 9.8%]). Overall, the proportion of deaths due to AIDS‐defining malignancy significantly reduced over time (Table 1).

Of 64 deaths due to non‐AIDS‐defining infections, most occurred in men (78%, 50/64) of White ethnicity (69%, 44/64) and 38% (24/64) occurred in people 65 years or older. Only three patients in this category died within 12 months of diagnosis, and seven had not had an ARV script within 6 months before death.

Cause of death was non‐AIDS‐defining malignancy in 113 people; 76% (86/113) were male, 68% (77/113) were >52 years old and 63% were in people of White ethnicity. Only 4.4% (5/113) died within 1 year of diagnosis, and only 10.7% (11/103) had a detectable viral load (>200 copies/mL) on last testing prior to death. The most common non‐AIDS‐defining malignancies were anal cancer (17, 15%), lung cancer (15, 13%), hepatocellular carcinoma (13, 12%) and haematological cancers (11, 10%).

Suicide, acute intoxication or chronic alcohol misuse were the cause of death in 49 people and, as a proportion of all deaths, increased from 5.3% (2006–2011) to 11.2% (2018–2023). Notably, in the context of the COVID‐19 pandemic, in 2021 and 2022, the proportion of deaths related to suicide or substance misuse was 16.7% (13/78). These deaths occurred predominantly in men (96%, 43/45) of White ethnicity (82%, 36/44). On average, the age at death for people in this group was 49 years, and time from diagnosis to death was 15 years. The majority of these patients were virally suppressed (87% <200 copies/mL, 40/46) and had received an ARV script within 6 months before death (81%, 34/42). Three deaths occurred within 1 year of diagnosis, and 14 had been seen by psychiatry or psychology teams prior to death. Of those who died by suicide, 5 of 18 had seen either psychology or psychiatry services between 2012 and 2023. Data on comorbidities, for example, substance misuse/dependency, was not available for all patients.

**FIGURE 2:**
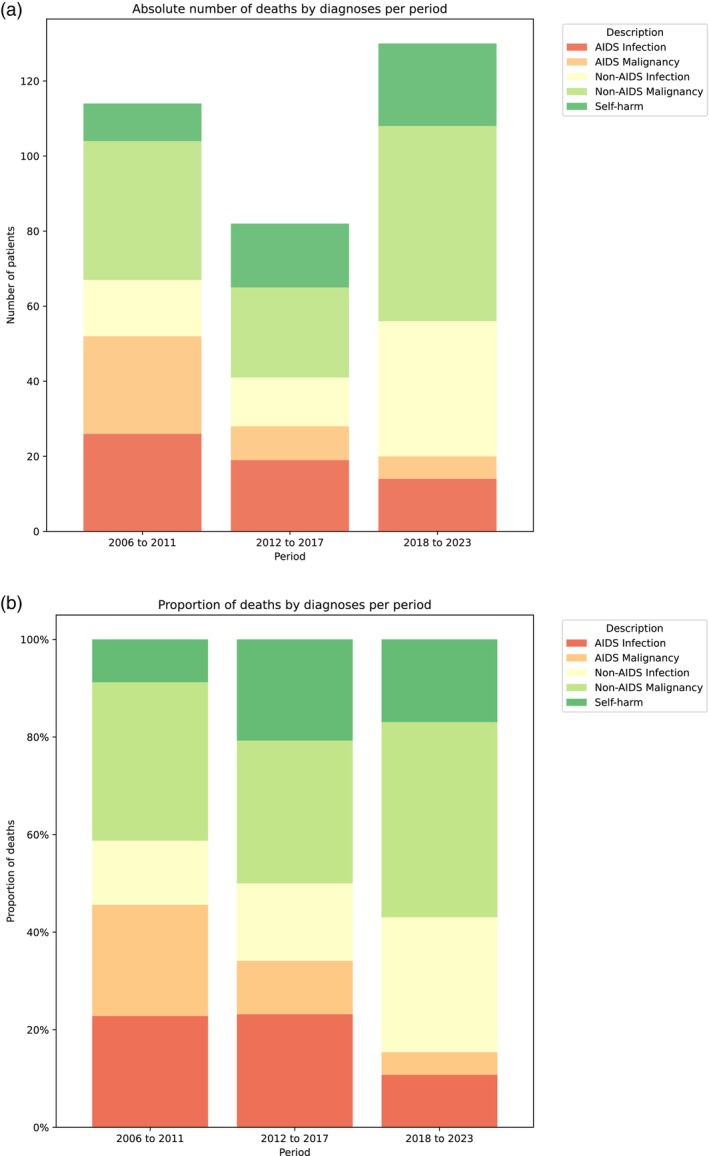
(a) Absolute number of deaths by diagnosis, 2026–2011, 2012–2017 and 2018–2023 and (b) Proportion of deaths by diagnosis, 2026–2011, 2012–2017 and 2018–2023.

AIDS‐defining causes of death, as a proportion of all causes of death, fell from 28% (52/188) between 2006 and 2011 to 10% (20/197) between 2018 and 2023; *p* < 0.05. Conversely, non‐AIDS‐defining causes of death increased (Figure 2). Non‐AIDS‐defining malignancy increased from 20% (37/188) between 2006 and 2011 to 26% (52/197) between 2018 and 2023. Non‐AIDS‐defining infection increased from 8% (15/188) to 18% (36/197) in the same time frames; however, COVID‐19 was the cause of death for 10 people in the 2018–2023 group.

### Demographics of the Royal Free HIV cohort

The Royal Free HIV cohort size in 2006–2011 was 3553 patients with a median age of 41.0 years compared with 3710 patients in 2018–2023 with a median age of 51 years; 74.0% were male in 2006–2011 compared with 72.4% in 2018–2023. Ethnicities, comparing 2006–2011 with 2018–2023, were: White British (38.3% vs. 32.8%), Black or Black British (26.5% vs. 25.8%), any other White background (19.3% vs. 19.1%), any other Black background (3.5% vs. 4.5%) and ‘other’ (10.9% vs. 14%).

## DISCUSSION

This longitudinal descriptive analysis provides insight into the changing epidemiology of causes of death in people with HIV over the past 18 years. The majority (79.8%) of deaths were in men, and almost 40% were in people of White British ethnicity, reflecting the demographic of people living with HIV who receive care in our centre. This is comparable to data reporting deaths among people accessing HIV care in the United Kingdom, where 76.4% and 77% of deaths were in men and 38.7% and 48.6% were in people of White British ethnicity in 2014 and 2023, respectively [7].

The median age at diagnosis and at death both increased gradually over time through our cohort, the latter increasing from 45 to 58 years old. The median time from diagnosis to death has also increased, reflecting improvements in linkage to care, overall medical and HIV care, and in particular the availability of increasingly effective and well‐tolerated ART. In our cohort, earlier diagnosis is supported by the fact that the CD4 count at diagnosis was higher in the latter part of the cohort, while the median viral load was lower. The rate of late diagnosis (CD4 < 350 cells/μL) remained high at 54% in the last 6 years of the cohort but had improved compared with 65% in the initial 6 years.

Only four of every five people received a script for ARVs within 6 months of death. This figure may be an underestimate as it does not account for other sources of ARVs, including other hospitals where people may have received inpatient care. However, it is evident that some deaths in this group were due to immunosuppression and inadequate treatment.

AIDS‐related deaths fell considerably over the 18 years, in keeping with the well‐established improvements in clinical care described previously. AIDS‐related deaths continue to disproportionately affect younger patients and those of Black ethnicity, calling for continued focus on early diagnosis and linkage to care. Although 83% had received an ARV script within 6 months, two‐thirds of people who died from an AIDS‐related cause had a detectable viral load, perhaps suggesting that these patients had not been on ARVs for long prior to death. Non‐AIDS‐defining infection occurred more frequently in the latter part of the cohort, even when excluding the 10 people who died due to COVID‐19; these tended to occur in older individuals, perhaps reflecting increased mortality from sepsis in this age group.

Non‐AIDS‐defining malignancies increased substantially and latterly were the leading cause of death in our cohort. Anal cancer, lung cancer and hepatocellular carcinoma were particularly prevalent, and these are likely to reflect additional risk factors in this group including HPV, HBV and HCV co‐infection and smoking. Data on these risk factors were inconsistently available in our cohort.

Deaths related to suicide, substance misuse or alcohol excess, as a proportion of all deaths, increased over time. A particularly sharp increase in these was seen in 2021–2022, possibly as a result of the impact of the global COVID‐19 pandemic on mental health, social isolation and reduced access to services.

Of our cohort, 3.4% died by suicide, compared with 0.97% in England and Wales according to Office for National Statistics data from 2022 [8]. A previous study of deaths attributable to suicide in people living with HIV in the United Kingdom found this to be the cause of death in 1.8% [9]. This study described higher rates of suicide in men compared with the general population, and in particular in the first year after diagnosis. In our cohort, all people whose death was attributable to suicide were men, supporting the notion that this group requires particular focus with regards to psychological support. However, only 11% died within 1 year of diagnosis, suggesting that this support needs to be sustained in the longer term. Data from the Poppy study suggests that in the United Kingdom and Ireland, over three quarters of people living with HIV who experience depressive symptoms did not report having access to any mental healthcare for these symptoms [10].

We were unable to ascertain the cause of death in 17.4% (92/529) of people. The proportion of deaths with an unknown or unclassifiable cause fell from 22.9% (43/188) in the first 5 years of the cohort to 12.2% (24/196) between 2018 and 2023. This is likely partly a reflection of less data loss through the use of electronic patient records compared with older paper records and possibly of improved engagement with care.

The national observational cohort of people living with HIV in England and Wales between 1997 and 2012 reported 9.3% of deaths with a missing cause [5]. Conversely, more recent studies show a trend towards higher proportions of missing data; 14% and 18% in audits of deaths in people with HIV in London in 2015 [11] and 2016 [2] respectively, 18% and 21% in the BHIVA/UKHSA National HIV Mortality Reviews of 2019 [12] and 2022 [13], respectively, and 21% in a recent descriptive study of the Swiss HIV cohort [14], which used the CoDe protocol to categorize the cause of death.

In our study, common reasons for missing information on the cause of death were that our service was notified of the death by someone with incomplete information (an individual's friend or family or by another clinic), that the death occurred abroad, that information from the outcome from a coroner's inquest was unavailable, and that our service did not have consent to contact other healthcare providers to collect further information.

While most people continue to die in hospital, the proportion of people dying at home increased over time. Trends were similar to the general population in England in 2022; hospital was the most common place of death (43.4%) although more than half of deaths (56.6%) occurred either at home (28.7%), in a care home (20.5%), in a hospice (4.7%) or in other places (2.6%) [15]. The increasing proportion of home deaths may reflect better accessibility and provision of palliative care and planning of death, but more prospectively collected information on end‐of‐life care would help better understand this trend and exclude barriers to access as a reason for these changes.

More work is required to better understand and target avoidable deaths in people with HIV. Future work should focus on systematic and prospective collection of lifestyle information and on early and proactive management of risk factors in people with HIV, including tobacco smoking, alcohol use and substance misuse, as well as identifying those with hypertension, hyperlipidaemia and diabetes mellitus. In the context of an ageing cohort, early detection and management of comorbidities is an increasingly important aspect of care. Despite marked improvements in overall mortality, people with HIV continue to have an increased risk of a non‐AIDS‐related death when compared with the general population. Our data suggest service delivery should focus on expanding access to co‐infection and cancer screening and prevention programmes, as well as smoking cessation, drug and alcohol support and mental health services to reduce excess preventable deaths in this group. It is fundamental that these services are available and that these comorbidities are regularly re‐assessed in the long term for people living with HIV, not only in the initial months after diagnosis. While AIDS‐related deaths have substantially reduced, the ongoing high rates of late HIV diagnosis suggest limited effectiveness of policies aimed at improving this thus far. More aggressive strategies, like ‘opt out’ testing in Emergency Departments, may help achieve progress in this regard.

The retrospective nature of our study leads to some important limitations, including ascertainment bias in classifying causes of death. Additionally, our analysis was limited to the available data, so we were unable to further explore areas of particular interest, including risk factor data. Although some lifestyle and specialty appointment information was available for a subset of people, it is not known whether they had access to services and the ability to modify their lifestyle choices and risk factors (with or without peer‐ or health care professional‐support), or the extent to which any comorbidities were controlled. Finally, the findings from this single centre study may not be representative of trends in causes of death in people with HIV outside London, or in similar large European cities, but data from national surveillance of people who access HIV care in the United Kingdom and recently published literature from similar settings suggest many similarities in the population studied and the trends observed.

The falling proportion of deaths attributable to advanced HIV, and a clear trend towards non‐AIDS malignancy and other non‐communicable diseases reflects much of the published literature from similar high‐income settings [14, 16, 17].

The most recently published national UK data on causes of death from Croxford et al. [5] provides useful, but aggregated, data on causes of death in people living with HIV between 1997 and 2012. By longitudinal data collection, our study permits analysis of recent trends in mortality in the United Kingdom. Compared with other recently published literature [14, 16, 17], this study adds value in that it reports a higher proportion of categorized causes of death with increased granularity. In particular, compared with other European and North American settings, it highlights seemingly unique UK trends of increasing suicide and self‐harm related causes of death, cardiovascular disease‐related causes of death and certain non‐AIDS malignancies, particularly anal cancer.

We highlight the current and ongoing need for earlier diagnosis of HIV, mental health support and early and proactive management of comorbidity and lifestyle risk factors to enable reduction in avoidable excess deaths in people with HIV, despite improvements in linkage to care and reduction in AIDS‐related deaths.

## AUTHOR CONTRIBUTIONS

RFM, LCV and DKM conceptualized the study. JH and AH curated the database. DKM performed the statistical analyses, and RFM, LCV and DKM analysed the data, with critical input from JH, AH and FMB. LCV, DKM and RFM wrote the first draft of the manuscript. All authors contributed to the writing of subsequent drafts and reviewed and approved the final manuscript. DKM, LCV and RFM verified the underlying data and jointly act as guarantors.

## CONFLICT OF INTEREST STATEMENT

The authors declare no conflicts of interest.
